# Combined Immunodeficiency Caused by a Novel Nonsense Mutation in *LCK*

**DOI:** 10.1007/s10875-023-01614-4

**Published:** 2023-12-19

**Authors:** Baerbel Keller, Shlomit Kfir-Erenfeld, Paul Matusewicz, Frederike Hartl, Atar Lev, Yu Nee Lee, Amos J. Simon, Tali Stauber, Orly Elpeleg, Raz Somech, Polina Stepensky, Susana Minguet, Burkhart Schraven, Klaus Warnatz

**Affiliations:** 1 Department of Rheumatology and Clinical Immunology, Medical Center - University of Freiburg, Faculty of Medicine, University of Freiburg, Freiburg, Germany; 2 Center for Chronic Immunodeficiency (CCI), Medical Center - University of Freiburg, Faculty of Medicine, University of Freiburg, Freiburg, Germany; 3 Department of Bone Marrow Transplantation and Cancer Immunotherapy, Hadassah Medical Center, Faculty of Medicine, Hebrew University of Jerusalem, Jerusalem, Israel; 4 Faculty of Biology, University of Freiburg, Freiburg, Germany; 5 Signalling Research Centres BIOSS and CIBSS, Faculty of Biology, University of Freiburg, Freiburg, Germany; 6 Pediatric Department A and the Immunology Service, Jeffrey Modell Foundation Center; Edmond and Lily Safra Children’s Hospital, Sheba Medical Center, affiliated to the Sackler Faculty of Medicine, Tel Aviv University, Tel-Aviv, Israel; 7 Department of Genetics, Hadassah, Hebrew University Medical Center, Jerusalem, Israel; 8 Health Campus Immunology, Infectiology and Inflammation (GC-I3) Medical Faculty, Otto-Von Guericke University Magdeburg, Magdeburg, Germany; 9 Center of Health and Medical Prevention (CHaMP), Otto-Von Guericke University Magdeburg, Magdeburg, Germany; 10 Department of Immunology, University Hospital Zurich, Zurich, Switzerland

**Keywords:** LCK, combined immunodeficiency, T cells, TCR signaling, low CD4 expression, diagnosis

## Abstract

**Abstract:**

Mutations affecting T-cell receptor (TCR) signaling typically cause combined immunodeficiency (CID) due to varying degrees of disturbed T-cell homeostasis and differentiation. Here, we describe two cousins with CID due to a novel nonsense mutation in *LCK* and investigate the effect of this novel nonsense mutation on TCR signaling, T-cell function, and differentiation.

Patients underwent clinical, genetic, and immunological investigations. The effect was addressed in primary cells and LCK-deficient T-cell lines after expression of mutated LCK.

**Results:**

Both patients primarily presented with infections in early infancy. The *LCK* mutation led to reduced expression of a truncated LCK protein lacking a substantial part of the kinase domain and two critical regulatory tyrosine residues. T cells were oligoclonal, and especially naïve CD4 and CD8 T-cell counts were reduced, but regulatory and memory including circulating follicular helper T cells were less severely affected. A diagnostic hallmark of this immunodeficiency is the reduced surface expression of CD4. Despite severely impaired TCR signaling mTOR activation was partially preserved in patients’ T cells. LCK-deficient T-cell lines reconstituted with mutant LCK corroborated partially preserved signaling. Despite detectable differentiation of memory and effector T cells, their function was severely disturbed. NK cell cytotoxicity was unaffected.

Residual TCR signaling in LCK deficiency allows for reduced, but detectable T-cell differentiation, while T-cell function is severely disturbed. Our findings expand the previous report on one single patient on the central role of LCK in human T-cell development and function.

**Graphical Abstract:**

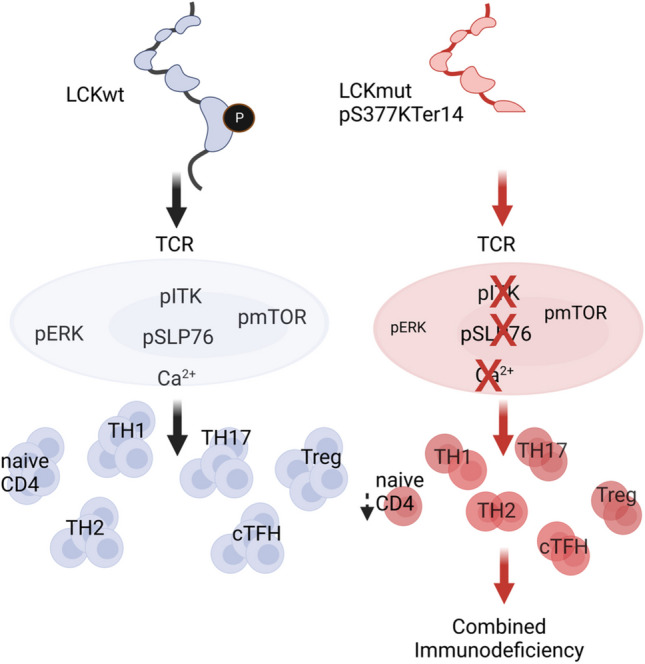

**Supplementary Information:**

The online version contains supplementary material available at 10.1007/s10875-023-01614-4.

## Introduction

The integrity of T-cell receptor (TCR) signaling is crucial for T-cell development, selection, differentiation and function, thus for the maintenance of cellular immunity. Monogenic variants in genes encoding for TCR signaling components result in inborn errors of immunity (IEI) with varying degrees of immunological impairment and a diverse spectrum of clinical phenotypes depending on the affected protein and the severity of the mutation [1, 2]. The loss of CD3δ, -ε or -ζ results in almost absent T-cell development [3], presenting as severe combined immunodeficiency (SCID) characterized by a profound susceptibility to opportunistic infections, failure to thrive, and death within the first 2 years of life unless the patients undergo hematopoietic stem cell transplantation (HSCT) [4]. In contrast, patients with mutations in TCR signaling components as *ZAP70*, *ITK, SLP76*, or *LAT* may have even normal numbers of peripheral T cells with yet altered function [5–8]. These combined immunodeficiencies (CID) are defined by increased susceptibility to opportunistic infections and carry a high incidence of morbidity and mortality. CID are also frequently associated with autoimmunity and other forms of immune dysregulation [1, 3].

TCR signaling is a well-regulated process. Upon activation of the TCR, active LCK initiates the phosphorylation of immunoreceptor tyrosine-based activation motifs (ITAMs) in the cytosolic tail of the CD3 chains, whereby the precise mechanism is still under debate [9–12]. The phosphorylated ITAMs serve as docking sites for the tandem SH2 domains of the tyrosine kinase ZAP-70, which is then recruited to the phosphorylated TCR complex. Subsequently, LCK also phosphorylates and activates ZAP-70 resulting in the presence of two active tyrosine kinases in the vicinity of the TCR. Upon phosphorylation of critical tyrosines, the LAT signalosome is assembled comprising the tyrosine kinase ITK, the adapter proteins Gads and SLP76, and the lipase PLCγ1, which is activated and which hydrolyzes PIP_2_ to generate IP3 and DAG. IP3 induces Ca^2+^ mobilization, and DAG activates the canonical NF-ĸB pathway, as well as the Ras/Raf/MAPK/AP1 pathway via activation of the upstream nucleotide exchange factor RasGRP [13], eventually leading to the activation of transcription factors and T-cell activation. Moreover, mTOR is activated, crucially affecting T-cell metabolism and differentiation [14].

The most upstream enzyme in TCR signaling is the Src family kinase LCK. Structurally, the N-terminal SH4 domain anchors LCK to the plasma membrane. LCK further comprises a unique region (UR), mediating binding to the co-receptors CD4 and CD8 as well as highly conserved regulatory SH3 and SH2 domains that are involved in the regulation of LCK’s conformation, activity, and interaction with other proteins [15]. The kinase domain contains a central tyrosine, Y394, crucial for kinase activity and a C-terminal negative regulatory tail carrying the tyrosine residue Y505. Phosphorylation of Y505 by CSK generates an intramolecular binding site for the SH2 domain of LCK, thereby inducing a conformational change that arrests LCK in a closed/inactive state [16]. Dephosphorylation by the phosphatase, CD45, results in non-phosphorylated, “primed” LCK [10, 17]. Full kinase activity is conveyed by auto- or trans-phosphorylation of Y394 within the kinase domain of LCK.

To date, only one patient carrying a homozygous mutation in *LCK* has been reported. The patient presented CID with low CD4 T cells, recurrent respiratory tract infections, and early-onset inflammatory and autoimmune manifestations including neutrophilic panniculitis skin lesion with necrosis and abscesses which was associated with vasculitis and dermal lymphohistiocytic infiltration [18, 19]. Here, we describe two patients bearing a novel homozygous nonsense mutation in *LCK*. The mutation leads to reduced expression of a truncated LCK protein lacking parts of the kinase domain and the two regulatory tyrosine residues. Both patients suffered from recurrent infections, presenting as CID, with a progressive loss of naïve T-cell subsets, oligoclonal T cells but detectable differentiation of regulatory T cells (Treg), circulating follicular helper (cTfh), T helper (TH)1, TH2, and TH17 cells. Our molecular studies suggest that despite severely diminished TCR signaling, the mutated LCK still allows residual TCR signal transduction both in patients’ cells and when reconstituted in LCK-deficient T-cell lines.

## Material and Methods

### Patients

Blood was collected from patients and controls after obtaining written informed consent by the donor or the parents and after approval by the Hadassah and Israeli Ministry of Health Ethical Review Boards. Beside the described LCK-deficient patients with mutations in *ITK* c. 1764C > G (male, 8 years old [20]), *LAT* c.268_269delGG (male, 8 years old [8] and male, 1.5 years old), *CARD11* c.del2839-3019 (f, 1 year old [21]), *ORAI1* c.271C > T (male, 18 years old [22], *MALT1* (m, 3 years old [23]), and *STIM1* (f, 1 year old [23]) were analyzed. Healthy controls were adult blood donors (*n* = 26). For TRG sequencing, data were compared to 4 pediatric controls (2 years old).

### Antibodies Used in This Study

For flow cytometry. CD3 (UCHT1) BV605, CD3 (UCHT1) PE-Cy7, CD3 (SK7) PerCp-Cy5.5, CD3 (UCHT1) AF700, CD4 (RPA-T4) PE-Cy7, CD4 (RPA-T4) BV421, CD8 (SK1) PerCp-Cy5.5, CD8 (SK1) APC-Cy7, CD8 (SK1) BV421, CD10 (Hi10a) BV605, CD16 (3G8) FITC, CD19 (HIB19) APC-Cy7, CD21 (Bu32) PE-Cy7, CD25 (BC96) BV421, CD27 (M-T271) BV421, CD28 (CD28.2) PerCP-Cy5.5, CD31 (WM59) BV605, CD38 (HIT2) PerCp-Cy5.5, CD45 (HI30) Pacific Blue, CD45RA (HI100) APC-Cy7, CD45RA (HI100) BV605, CD56 (HCD56) AF700, CD127 (A019D5) APC, TCR αβ (IP26) PerCP-Cy5.5, TCR γδ (B1) APC, IL-2 (MQ1-17H12) PerCpCy5.5 TNF-α (Mab11) APC-Cy7 (BioLegend Inc., San Diego, CA); CD3 (UCHT1) BUV395, CD8 (M-T411) APC, CD8 (SK1) Pacific Blue, CD27 (L128) BUV395, CXCR5 (RF8B2) FITC, IFNγ (B27) FITC, IgG (G18-145) Alexa Fluor 700, ERK1/2(pT202/pY204) ( 20A) AF647, LCK N-terminal (MOL171) PE, SLP76(pY128) (J141-668.36.58) (BD Biosciences, Heidelberg, Germany); CD8 (SK1) PE, CD45RA (ALB11) FITC (Beckman Coulter GmbH, Krefeld, Germany); goat anti-human IgD FITC, goat anti-human IgA PE (Southern Biotech, Birmingham, AL); CXCR3 (49801) PE (R&D Systems, Minneapolis, MN); IL-17 (eBio64DEC17) PE, IL-4 (MP4-25D2) APC, BTK/ITK(pY551/pY511) (M4G3LN) PE, mTOR(pS2448) (MRRBY) PE, mouse IgG1 ĸ isotype PE (eBioscience Inc, San Diego, CA); goat anti-human IgM AF647 (Jackson Immunoresearch Laboratories, Suffolk, UK); LCK C-terminal (73A5) UNLB (Cell Signaling Technology, Leiden, The Netherlands).

The analysis of TCR Vb expression by flow cytometry was determined according to manufacturer’s manual (Beta Mark TCR Vb Repertoire Kit, Beckman Coulter) using the appropriate antibodies.

For western blot. LCK(pY192) (LS-C199194), LSBio, LCK(pY505) (2751S), Src(pY416) (6943S), ZAP70(pY319) (2701S), LAT(pY191) (3584S), SLP76(pS376) (14745S), ERK1/2 (pT202/pY204) (9101L) (Cell Signaling Technology, Leiden, The Netherlands); C-terminal LCK (3A5) (Santa Cruz); 4G10, (clone BE0194) (InVivoMab); ß-Actin (clone A5441), Sigma.

### TRG Repertoire Analysis

Genomic DNA from patients’ peripheral blood and skin biopsy and from age matched healthy pediatric controls (*n* = 4, 2 years of age) were used to generate TRG receptor repertoire according to the manufacturer’s protocol (LymphoTrack; Invivoscribe Technologies). The libraries were quantified and equimolar amounts were pooled and sequenced using Mi-Seq Illumina technology (Illumina). Sequencing results were converted to FASTA format (Geneious, Biomatters) and submitted to the IMGT HighV-QUEST webserver (http://www.imgt.org). Next, the statistics file from IMGT HighV-QUEST was used to generate hierarchial treemaps (Treemap software, Macrofocus GmbH). Shannon’s H and Simpson’s D diversity indices were calculated (PAST program; http://priede.bf.lu.lv/ftp/pub/TIS/datu_analiize/PAST/2.17c/ download.html). For the graphical presentation, Excel and Prism9 (GraphPad Software Inc., USA) were used. For the statistical analysis of top100 clones, one tailed *t*-test with Welch’s correction was used, and Gaussian distribution was assumed.

### Transient Transfection of Jk.LCKKO Cells and Generation of Inducible LCKmut Expressing T-Cell Lines

The LCK-deficient Jurkat variant Jk.LCKKO was kindly provided by Prof. Dr. Arthur Weiss (UCSF, CA, USA). For re-expression of LCKwt or LCKmut, 2 × 10^7^ Jk.LCKKO cells were transfected with 20 µg plasmids coding for LCKwt or LCKmut (both untagged) together with pEGFP_neo (to discriminate transfected cells from untransfected cells) using a Biorad Genepulser in 1 ml of RPMI 10% FCS and Penstrep. After transfection, the DNA was removed, and the transfected cells were incubated overnight in standard culture medium prior to further analysis.

Inducible LCKmut^−^expressing T-cell lines were generated as follows. The plix402_hLCKWT plasmid containing a tetracycline-controlled transactivator, a tetracycline-responsive promoter, and the coding sequence for wild-type human LCK were a generous gift from Oreste Acuto (UK). The kinase inactive mutant K273A and the S377KTer14 truncated form (LCKmut) were generated by mutagenesis PCR. Lentiviral particles were produced by co-transfection of plix402_hLCK constructs, pCMVDR8.74, and pMD2G plasmids using PEI (Polysciences) in HEK293T cells. Viruses were concentrated by centrifugation and used to transduce Jk.LCKKO cells. Briefly, 3 × 10^6^ cells per condition were cultivated in RPMI 5% Tet system-approved FCS, and the lentiviruses were added in the presence of 5 μg/ml of Polybrene. Cells were incubated overnight at 37 °C. On the next day, medium was exchanged. LCK expression was induced by incubation with 3 μg/ml doxycycline for 24 h at 37 °C. Induced expression of LCK was assayed by intracellular flow cytometry. Briefly, cells were fixed and permeabilized using BD Cytofix/Cytoperm kit. Cells were incubated with a rabbit anti-LCK antibody recognizing the N-terminal of LCK (overnight) and next day with a goat anti-rabbit IgG-Dylight 633 antibody for 30 min before analyzing them in a Cyan ADP Flow cytometer (Beckman Coulter).

### Western Blot

Jk.LCKKO cells transiently transfected with LCKwt or LCKmut were stimulated with 10 µg/ml anti-CD3 (UCHT1) for different periods of time at 37°. After stimulation, 1 ml ice cold PBS was added, and cells were briefly spun down and lysed for 20 min at 4 °C in 90 µl lysis buffer (1% NP40, 1% Lauryl-Maltoside, 1 mM Na-vanadat, 1 mM PMSF, 10 mM NaF, 10 mM EDTA, 50 mM TRIS, 150 mM NaCl2). Nuclei were removed by centrifugation (10 min, 13.000 rpm, 4 °C). The post-nuclear supernatant was supplemented with 22.5 ul of reducing 5 × sample buffer (50% Glycerol, 5% SDS, 10% ß-Mercaptoethanol, 0.25% Bromphenolblue) and boiled for 5 min at 100 °C. Twenty-five microliters of the suspension/lane was subjected to SDS-PAGE and Western-blotting onto Nitrocellulose membranes (Amersham). After incubation with primary antibodies, detection was achieved by using either peroxidase-conjugated affiniPure Goat-anti-mouse IgG + IgM (H + L, Dianova) or peroxidase-conjugated AffiniPure goat-anti-rabbit IgG (H + L, Dianova) and subsequent ECL (SIGMA, RPN2106) detection.

### Detection of Intracellular Signaling by Flow Cytometry

For detection of intracellular signaling proteins in primary T cells, fresh or thawed PBMCs were rest overnight at 37 °C. PBMCs were incubated with 5 µg/ml anti-CD3 (OKT3) (Biolegend) for 15 min on ice. Five microgram per milliliter goat-anti-mouse IgG was added for additional 15 min. For detection of phosphorylated mTOR and SLP76 cells were stimulated with anti-CD3 for 10 min at 37°, and for phospho-ITK and -ERK, cells were stimulated for 90 s. Phospho-AKT and -S6 were determined after 15 min. For some experiments, the LCK inhibitor A770041 was added 60 min prior to stimulation. PBMCs were fixed and permeabilized using the BD Biosciences®Phosflow intracellular staining kit following the manufacturer’s instructions. Staining for CD3, CD4, and CD45RA was performed for gating on naïve CD4 T cells.

Ca^2+^ mobilization in PBMCs was determined as described [8, 24]: cells were labeled with 4.5 µM Indo-1 AM/ 0.045% Pluronic F-127 (both Thermo Fisher) for 45 min, washed twice with IMDM 10% FCS, and stained for CD45RA, CD8, CD4, CD28, CD27, and TCR γδ for 15 min at room temperature. After washing 0.6 µg/ml anti-CD3 (UCHT) was added 10 min prior to data acquisition. Baseline Ca^2+^ levels were recorded for 45 s, before crosslinking of anti-CD3 antibodies with 5 μg/ml goat anti-mouse IgG (Jackson ImmunoResearch Laboratories, Inc.). Ca^2+^ measurements of transiently transfected Jk.LCKKO cells were determined with minor changes: after incubation of 0.5 × 10^6^ cells/sample in phenol red-free RPMI (Gibco) 10% FCS and 5 µg/ml Indo-1 AM, a tenfold volume of RPMI 10% FCS was added, and the cells were incubated for additional 45 min at 37 °C. Cells were then spun down and resuspended in phenol red-free RPMI. The baseline of the unstimulated cells was recorded for 1 min, and cells were stimulated with culture supernatant of the monoclonal anti-TCR antibody C305 (1:100) (IgM, kindly provided by Prof. Arthur Weiss). Ca^2+^ mobilization experiments were measured with a BD LSRFortessa cell analyzer (BD Bioscience) equipped with a 355-nm laser. Iononomycin was added as a positive control at a final concentration of 1 µg/ml.

In stably transduced T-cell lines, LCK expression was induced as described above and signaling determined as follows. TCR stimulation was performed at 37 °C with 3 μg/ml of the anti-human CD3ε antibody OKT3 for the indicated times or left unstimulated. Cells were fixed and permeabilized using BD Cytofix/Cytoperm kit, and intracellular staining was performed as described above using Erk1/2 (pT202/Y204). Goat anti-rabbit IgG-Dylight 633 antibody was used before analyzing them in a Gallios Flow cytometer (Beckman Coulter). To determine Ca^2+^ mobilization, 10^6^ cells were suspended in medium containing 1% Tet System approved FCS and incubated for 45 min at 37 °C in the dark with Fura red (Thermo Fisher Scientific) and Fluo-3 (Invitrogen). Cells were then washed and mixt with pre-warmed medium just before recording in a Cyan ADP Flow cytometer (Beckman Coulter). After 1 min recording, 3 μg/ml of the stimulating antibody OKT3 was added and recording continued for another 5 min.

### Cytokine Production and Functional Assays

Intracellular cytokines were determined after stimulation of PBMCs with 5 ng/ml PMA and 0.75 µg/ml ionomycin or 10 µg/ml anti-CD3 (OKT3) and 2 µg/ml anti-CD28 in the presence of 10 µg/ml Brefeldin A (Sigma-Aldrich) as described before [8]. T-cell activation and T-cell proliferation after labeling of PBMCs with 0.5 µM CFDA/SE were determined after stimulation with 0.5 µg/ml and 1 µg/ml anti-CD3, respectively, and 1 µg/ml CD28 [8, 25] NK cell degranulation was performed as described before [8, 25].

## Results

### Clinical Presentation

Two children, second-degree cousins born to a highly consanguineous family, were evaluated. Patient I (Pat I) is a female, born to first degree consanguineous parents. The patient was born after normal pregnancy and delivery and uneventful perinatal course. At the age of 6 months, she started to suffer from recurrent pneumonia and otitis media with recurrent skin infections of unknown nature. At 11 months, she was treated for severe chest infection requiring mechanical ventilation and transferred to our care for immunological work up. During the hospitalization, hypogammaglobulinemia was noted at the age of 13 months. She received HSCT from her fully matched (10/10) heterozygous father following Fludarabine, Treosulfan, Thiotepa, and ATG conditioning. Due to donor being hepatitis B positive, the patient received prophylactic treatment with immunoglobulin against hepatitis B from day 0 till + 7 and was treated prophylactically with lamivudine for 6 months. Now, she is 4.5 years after transplantation with 20% donor chimerism, without any infections, attending school.

Patient II (Pat II) was evaluated at another hospital and was transferred to our care for HSCT. Since infancy, she was reported to have failure to thrive, recurrent chest and ear infections, positive stool for cryptosporidium antigen, and oral candidiasis. Her peripheral blood was consistently positive to both CMV (10^3^ copies/ml) and EBV (10^5^ copies/ml). In addition, she had recurrent erythematous and ulcerative plaque skin lesions over her body (Supplementary Fig S1) which were cultured negative for bacteria or fungus. She was treated with broad spectrum antibiotics and ganciclovir with relatively good response (CMV copies became undetectable). Blood cultures were repeatedly positive for methicillin-resistant *Staphylococcus aureus* (MRSA). Positron emission tomography (PET) scan demonstrated multiple cutaneous and subcutaneous lesions, and therefore another skin biopsy was requested. This time, it revealed fragments of skin showing slightly hyperplastic epidermis and subepidermal edema. The whole dermis and subcutis were infiltrated by patchy and nodular aggregates of CD68^+^ histiocytes and small mostly CD8-positive lymphocytes without evidence of atypical cells. The Ki67 proliferation antigen was positive in 20–30% of the cells. The findings were interpreted as a reactive granulomatous infiltrative process without detectable causative pathogen. Treatment with systemic steroids, cyclosporine and mycophenolate mofetil, however, induced only a partial response, leaving the question whether this was an autoimmune manifestation unanswered. The patient was placed on IVIG replacement therapy for newly diagnosed hypogammaglobulinemia and trimethoprim–sulfamethoxazole and fluconazole prophylaxis. At the age of 3.5 years, she was transplanted using a mismatched 9/10 unrelated donor following conditioning with Fludarabine, Treosulfan, and ATG. She was successfully engrafted but on day + 10 she developed a septic shock with blood cultures positive for *Candida glabrata* and vancomycin-resistant enterococci (VRE), to which she succumbed on day + 20.

The heterozygous parents of both patients did not show any clinically relevant symptoms.

### Genetic and Molecular Analysis

**Fig. 1:**
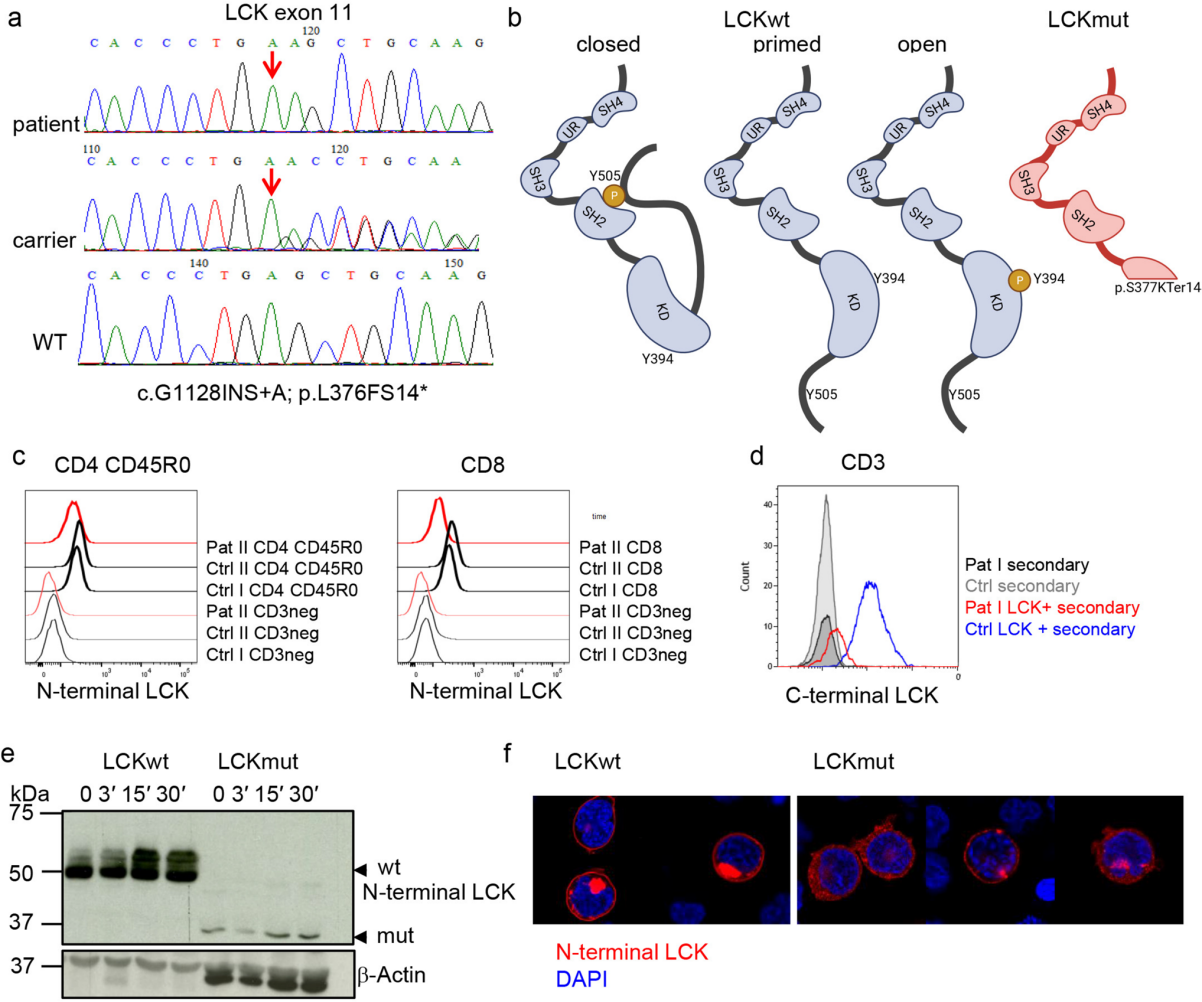
LCK expression. **a** Sanger sequencing of exon 11 in one patient, one heterozygous carrier and wild type sequence. Insertion is marked by a red arrow. **b** Schematic structure of wild type LCK protein and the different inactive or relevant active forms and predicted mutated LCK leading to a truncation of the kinase domain (red). Crucial tyrosine residues Y394 and Y505 are indicated. SH (SRC homology domain), UR (unique region), KD (kinase domain). **c** Histograms of LCK expression as determined by flow cytometry using an antibody targeting the N-terminal region LCK in CD4 CD45R0 T cells (left) and CD8 T cells (right) in Pat II (red) and two healthy controls (ctrl, black) (bold lines) compared to LCK negative CD3neg non-T cells (thin line). **d** Flow cytometric analysis of LCK, targeting the C-terminal region in CD3 T cells from Pat I (red) and one ctrl (blue). Secondary antibody staining is indicated in black (Pat) and grey (Ctrl), respectively. **e** Western blot of LCK-deficient Jk.LCKKO transduced T-cell lines with wild type LCK (LCKwt) and mutated LCK (LCKmut) unstimulated and after anti-CD3 stimulation as indicated targeting the N-terminal region of LCK. **f** Localisation of LCK (shown in red) in Jk.LCKKO transfected with LCKwt or LCKmut. DAPI staining is shown in blue

Whole exome sequencing revealed a homozygous single nucleotide insertion in *LCK* (chr1-32,745,529 InsA, NM_005356.5, c.1129dupA Ser377fs rs1569967422) in both patients, resulting in a frameshift and a premature stop codon at position c.1171 of the *LCK* transcript. The mutation was confirmed by Sanger sequencing with full family segregation (Fig. 1a). The mutation suggested the expression of a truncated LCK protein (p.S377KTer14, LCKmut), with an incomplete kinase domain, lacking the crucial tyrosine residues Y394 and Y505 (Fig. 1b). Flow cytometric analysis with an antibody targeting the N-terminal part of the protein revealed reduced expression of LCK in patient’s CD4 CD45R0 and CD8 T cells compared to healthy controls (Fig. 1c and Supplementary Figure S2a for control staining). In line with the prediction of a truncated LCK version, an antibody against the C-terminal end of LCK failed to detect the protein in patient’s T cells (Fig. 1d). Transient expression of LCKmut in LCK-deficient Jurkat cells (Jk.LCKKO) [26] resulted in reduced expression of a shortened protein compared to LCK wild-type (LCKwt) transfected cells (Fig. 1e). Microscopic analysis showed an aberrant subcellular localization of the truncated LCK in T-cell lines compared to the wild type protein (Fig. 1f and Supplementary Figure S2b for controls).

### Immune Phenotype

**Fig. 2:**
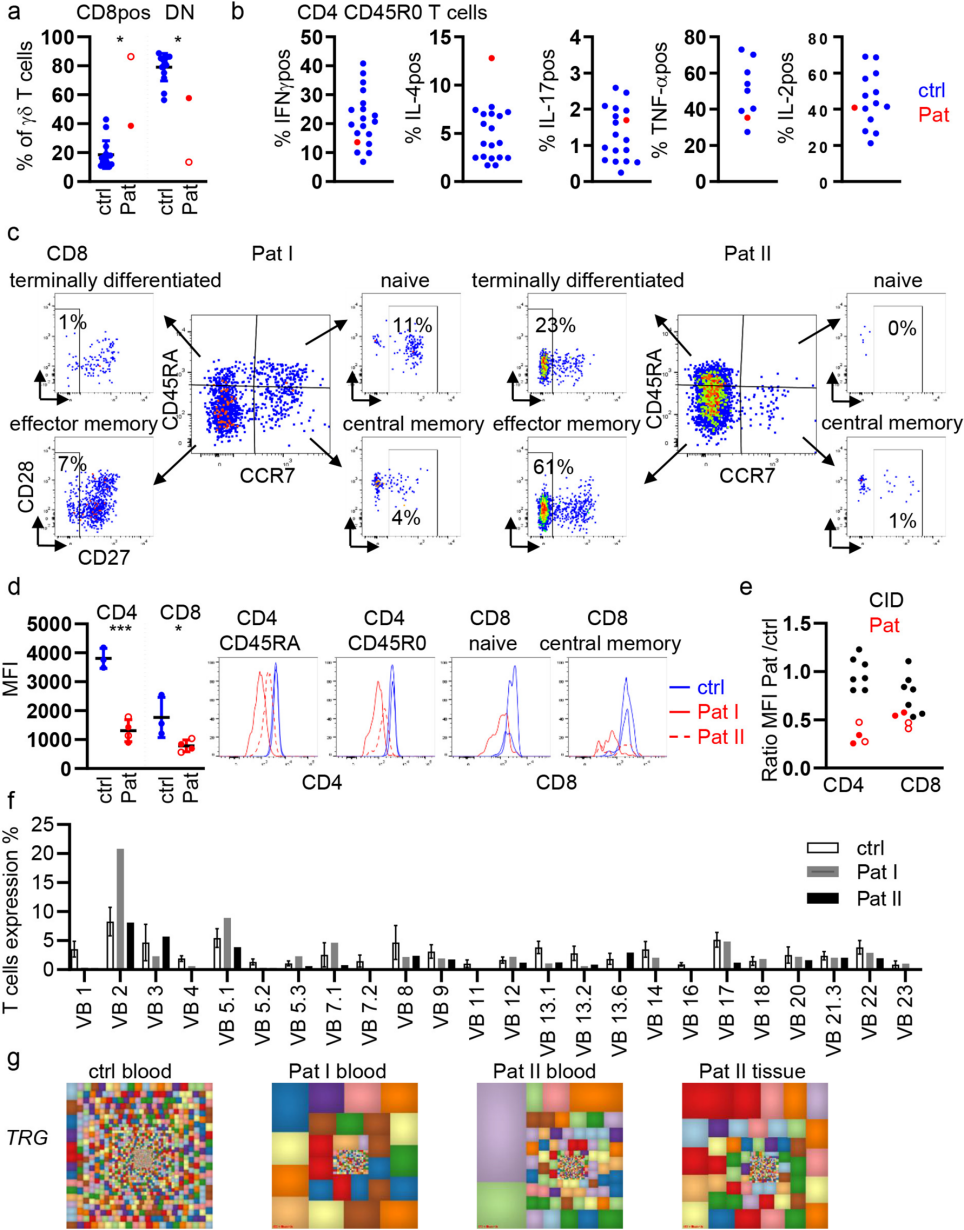
T-cell phenotype in LCK-mutated patients. **a** Proportion of CD8pos and CD4/CD8 double negative (DN) γδT cells in patients with *LCK* mutation (Pat I filled, Pat II open circle) and 8 controls (ctrl). **b** Percentage of IFNγ, IL-4, IL-17, TNFα and IL-2 positive CD45R0/CD4 T cells. IL-4 positive cells were determined after incubation with BfA; all other cytokines refer to PMA/Ionomycin (P/I) stimulated cells. **c** CD8 T-cell subsets in Pat I and II as referred to Table 1. **d** MFI of CD4 and CD8 in CD4 and CD8 T cells of Pat I and II and 3-day controls (ctrl) and histogram overlays of distinct subsets in Pat I (red solid line) and Pat II (red dotted line) and 2-day controls (blue). **e** Ratio of the MFI of CD4 and CD8 in patients with CID due to mutations in ITK, LAT, CARD11, MALT1, STIM1, and ORAI1 (black) and in LCK-deficient patients Pat I (filled red dots, *n* = 2) and Pat II (open red dots, *n* = 2). **f** TCR Vβ gene families in controls (white bars, *n* = 85), Pat I (grey) and Pat II (black bars). **g** Hierarchical Treemaps graphically representing the overall TRG repertoire from peripheral blood of one representative pediatric control and both patients and skin biopsy of one patient

Extended immune phenotypic evaluation was performed at the age of 15 (patient I) and 30 (patient II) months of age. Both patients exhibited reduced absolute lymphocyte counts, reduced absolute and relative CD3, CD4, and CD8 T-cell counts, reduced T-cell receptor excision circles (TRECs), and increased proportions of γδT cells (Table 1). Overall, the observed changes as well as a shift within γδT cells towards CD8^pos^ TCRγδT cells were more pronounced in patient II, probably due to a concurrent CMV reactivation (Fig. 2a and see Supplementary Figure S2c for gating strategy).

**Table 1:** Immunephenotype

		Pat I (15 m)	Age-matched reference values	Pat II (42 m)	Age-matched reference values
Lymphocytes/µl		1,400	3200–12,300	600	1400–5500
CD3 T cells (% of lymphocytes)		23.4	56–87	11.5	52–92
CD3 T cells /µl		328	2400–8300	69	850–4300
CD4 T cells (% of lymphocytes)		16.2	25–86	4.8	25–66
CD4 T cells /µl		227	1300–7100	29	500–2700
CD8 T cells (% of lymphocytes)		3.9	7–58	5.7	9–49
CD8 T cells /µl		54	400–4100	34	200–1800
γδT cells of CD3		14.9	< 10	37.0	< 10
B cells (% of lymphocytes)		43.4	3–77	75.7	8–39
B cell /µl		3226	110–7700	454	180–1300
NK cells (% of lymphocytes)		22.7	1–64	10.9	2–25
NK cells /µl		318	71–3500	65	61–510
TREC copies		3.5	> 400	8	> 400
CD4 subpopulations	CD45RA	26.5	82.3–95.1	2.3	71.5–84.2
	RTE (CD31 + of CD45RA)	71.8	48.4–76.3	52.1	65–79.5
	Treg (CD127-CD25 + +)	3.9	3.7–6.7	4.5	2.9–7.4
	cTFH (CXCR5 + of CD45RA −)	29.3	18.4–29.9	32.4	18.4–29.9
CD8 subpopulations	Naive (CD45RA + /CCR7 + /CD27 +)	10.6	16–100	0	19–100
	effector memory (CD45RA-/CCR7-/CD27-)	7.2	1–100	60.7	10–55
	central memory (CD45RA-/CCR7 + /CD27 +)	3.4	2–6	0.6	1–9
	terminally differentiated (CD45RA + /CCR7-/CD27-)	1.5	4–92	22.9	6–83
B cell subpopulations	Transitional			2.7	6.4–13.9
	Naive (CD27-/IgD + /CD38 + /CD10-)			85.9	49.7–77.1
	IgM memory (CD27 + /IgD +)			2.6	4.8–16.1
	IgM only (CD27 + /IgD-/IgM +)			1.6	0.9–9.3
	switched memory IgA (CD27 + /IgA +)			0.8	1.0–3.5
	switched memory IgG (CD27 + /IgG +)			0.6	1.8–6.2
	CD21low (CD21-CD38-)			0.6	
	Plasmablasts (CD38 + + /CD27 +)			0.04	

Absolute numbers refer to number of cells per µl. If not mentioned differently, relative counts are shown as percentage of parental population. *TRECs*, T-cell receptor excision circles; *RTE*, recent thymic emigrants; *Treg*, regulatory T cells; *cTFH*, circulating T follicular helper cell. Reference values were taken from [27, 28] or were internal reference values

Further analysis revealed a severe loss of naïve T cells especially in patient II (Table 1). Interestingly, the proportions of Treg and cTFH T cells were normal (Table 1). Similarly, normal cytokine production upon stimulation with PMA/Ionomycin demonstrated a regular in vivo differentiation of TH1 and TH17 as well as IL-2 and TNF-α-producing T cells and an increased proportion of IL-4-producing TH2 cells (Fig. 2b). The distribution of central memory, effector memory and terminally differentiated CD8 T cells did not allow to detect a common pattern between both siblings probably due to the effects of the concomitant CMV infection in patient II (Table 1 and Fig. 2c).

As previously published for the first patient with a *LCK* mutation and a murine *lck* knock out model [18, 29], the expression of both co-receptors CD4 and CD8 was significantly diminished in CD4 and CD8 T cells from patients compared to controls (Fig. 2d). CD4 expression was reduced in all patient-derived CD4 T-cell subsets, but CD8 expression was less consistently diminished, as shown by almost normal expression in very small subsets of naïve and central memory CD8 T cells (Fig. 2d). Expression of CD4 and CD8 was further addressed in other patients with CID due to mutations in TCR signaling components (LAT, ITK, ORAI1, MALT1, CARD11, STIM1) [8, 21, 30, 31] and unpublished) and the respective controls. The low CD4 expression was exclusive to LCK deficiency, while low expression of CD8 could also be observed in other forms of CID (Fig. 2e).

Absolute B-cell- and NK-cell counts were normal in both patients. B-cell subset analysis in patient II showed reduced IgG^pos^ switched memory B cells (Table 1), presumably resulting from insufficient T-cell help.

Analysis of TCR Vb expression showed an oligoclonal repertoire with the expansion of few and the absence of some Vb subfamilies in both patients (Fig. 2f). Also sequence analysis of the *TRG* locus demonstrated an oligoclonally restricted immune repertoire of TCRγδ T cells in blood of both patients and from skin biopsy of patient II as visualized by large uneven squares in the treemaps, where each square represents a unique recombination/sequence and the size of the square display the abundance (Fig. 2g). This corresponded to the distinctly higher calculated Simpson’s D index of unevenness observed in the repertoire of the peripheral blood of both patients and skin biopsy for patient II compared to pediatric controls (Supplementary Fig. S3a). Furthermore, the frequencies of the top 100 most abundant clones were significantly higher in the peripheral blood of both patient I and patient II compared to healthy pediatric controls (Supplementary Fig S3b).

To quantify the diversity, we calculated the Shannon’s H diversity index which takes into account both the unique and total number of sequences. Corresponding to the treemaps (Fig. 2g), both patients showed markedly lower Shannon’s H index compared to pediatric controls (Supplementary Fig S3c). Lastly, we analyzed the *TRGV* gene usages both in unique and total sequences of peripheral blood and skin biopsy. The data showed a unique pattern in the TRG repertoire with profoundly utilized *TRGV8* usage but lower *TRGV10* usage for both patients compared to controls (Supplementary Fig. S3d and e), which may be due to both primary and secondary effects of the *LCK* mutation.

### Signaling Capacity of Mutated LCK in Primary T Cells

**Fig. 3:**
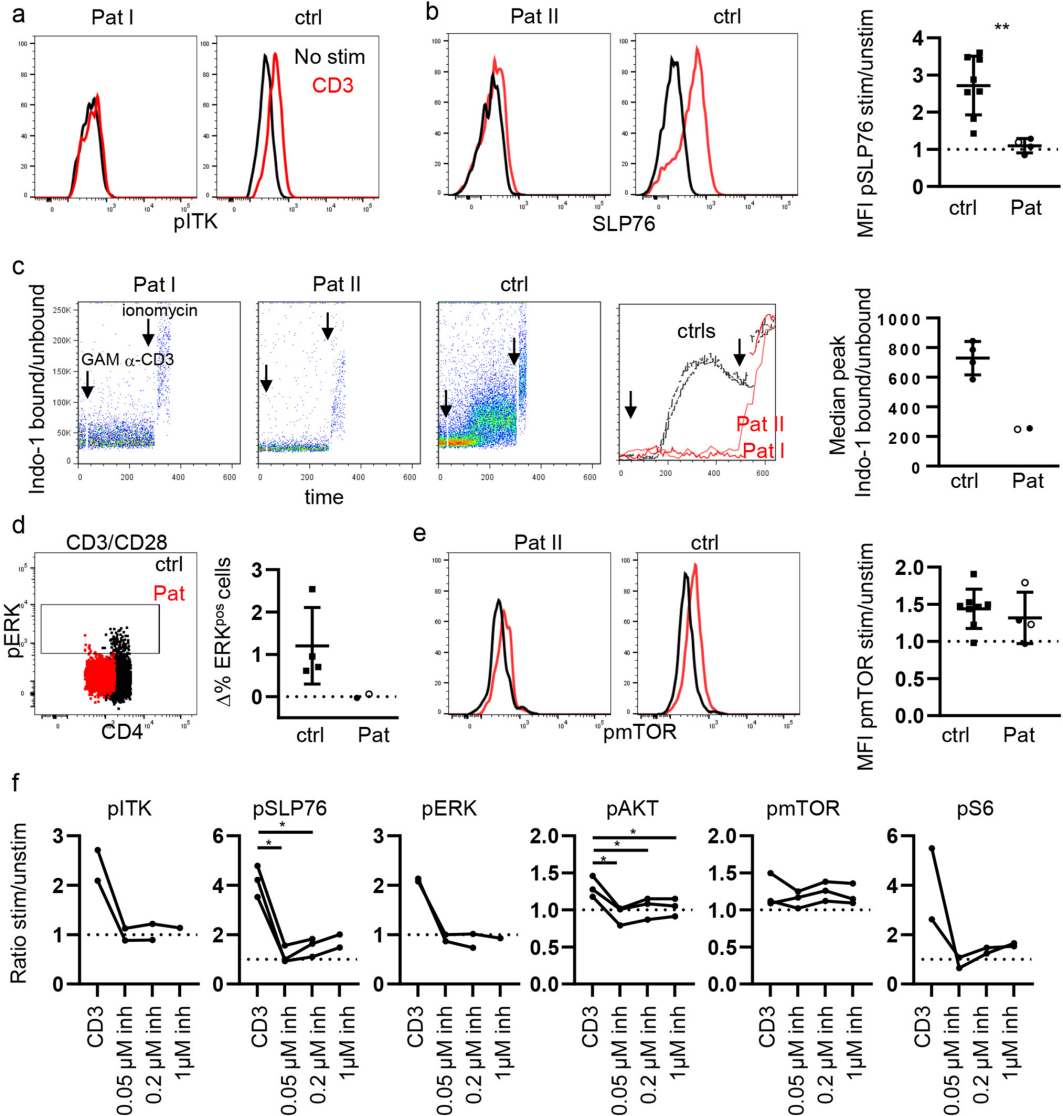
TCR signaling in primary T cells of patients. **a** Histograms of pITK in T cells from Pat I and the respective day control unstimulated (red) and after stimulation with anti-CD3 (red). **b** Representative histogram overlay of pSLP76 in CD45RA/CD4 T cells from Pat II and control unstimulated (black) and after stimulation with anti-CD3 (red). Statistics of the MFI stim/unstim of pSLP76. Pat I filled circle, three independent experiments. Pat II open circle. **c** Ca^2+^ mobilization in CD45RA/CD4 T cells after crosslinking of anti-CD3 with goat anti-mouse IgG (GAM) as determined by Indo-1 AM. Addition of GAM and ionomycin is indicated by arrows in Pat I, Pat II, and one control and kinetics overlay. Quantification of the peak median fluorescence intensity Indo-1 bound/unbound after GAM crosslinking. **d** Representative dot plot of pERK^pos^ CD45RA/CD4 T cells after anti-CD3/CD28 stimulation. Statistics depict the difference of pERK^pos^ stim-unstim in patients and four controls. **e** Representative histogram overlays of pmTOR in Pat II and the respective control stimulated (red) and unstimulated (black) after stimulation with anti-CD3. Statistics of the MFI of pmTOR stim/unstim in patients and 8 controls. Each patient was analyzed twice. **f** Ratio of the MFI anti-CD3 stim/unstim of phosphorylated ITK, SLP76, ERK, AKT mTOR, and S6 determined after incubation of PBMCs with the LCK inhibitor A770041 in naïve CD4 T cells from healthy donors. *p < 0.05, **p < 0.01

We next analyzed TCR signaling in primary CD4 T cells of both patients to further characterize the impact of the novel *LCK* mutation. To exclude confounding factors due to the differential T-cell subset composition, the analysis was restricted to naïve CD45RA CD4 T cells. Phosphorylation of ITK was not detectable in T cells from patient I after stimulation with anti-CD3 (Fig. 3a). Likewise, phosphorylation of SLP76 was significantly reduced in both patients’ T cells compared to the respective controls (Fig. 3b). Corroborating defective TCR proximal signaling, mobilization of Ca^2+^ upon anti-CD3 crosslinking could not be detected in primary T cells of both patients (Fig. 3c), and ERK phosphorylation was reduced after stimulation (Fig. 3d). In contrast, phosphorylation of mTOR seemed comparable to controls (Fig. 3e). Overall, these data suggest strongly impaired signaling downstream of the TCR, while preserved mTOR phosphorylation is compatible with residual signaling function of the truncated LCK protein or LCK-independent phosphorylation of mTOR. To address this question, primary CD4 T cells of healthy controls were stimulated with anti-CD3 after treatment with various concentration of the specific LCK inhibitor A770041. In line with patients’ T cells, the presence of the LCK inhibitor strongly diminished the phosphorylation of ITK, SLP76 and ERK, whereas phosphorylation of mTOR was not affected (Fig. 3f). The phosphorylation of AKT and S6, however, was significantly reduced by pre-treatment with the inhibitor (Fig. 3f). Altogether, our data from the patients and pharmacological inhibition support a scenario where mTOR is phosphorylated at S2448 independently of LCK catalytic activity and the classical TCR-LCK-AKT-mTOR-S6 signaling pathway in human primary CD4 T cells.

### Lymphocyte Activation and Function

**Fig. 4:**
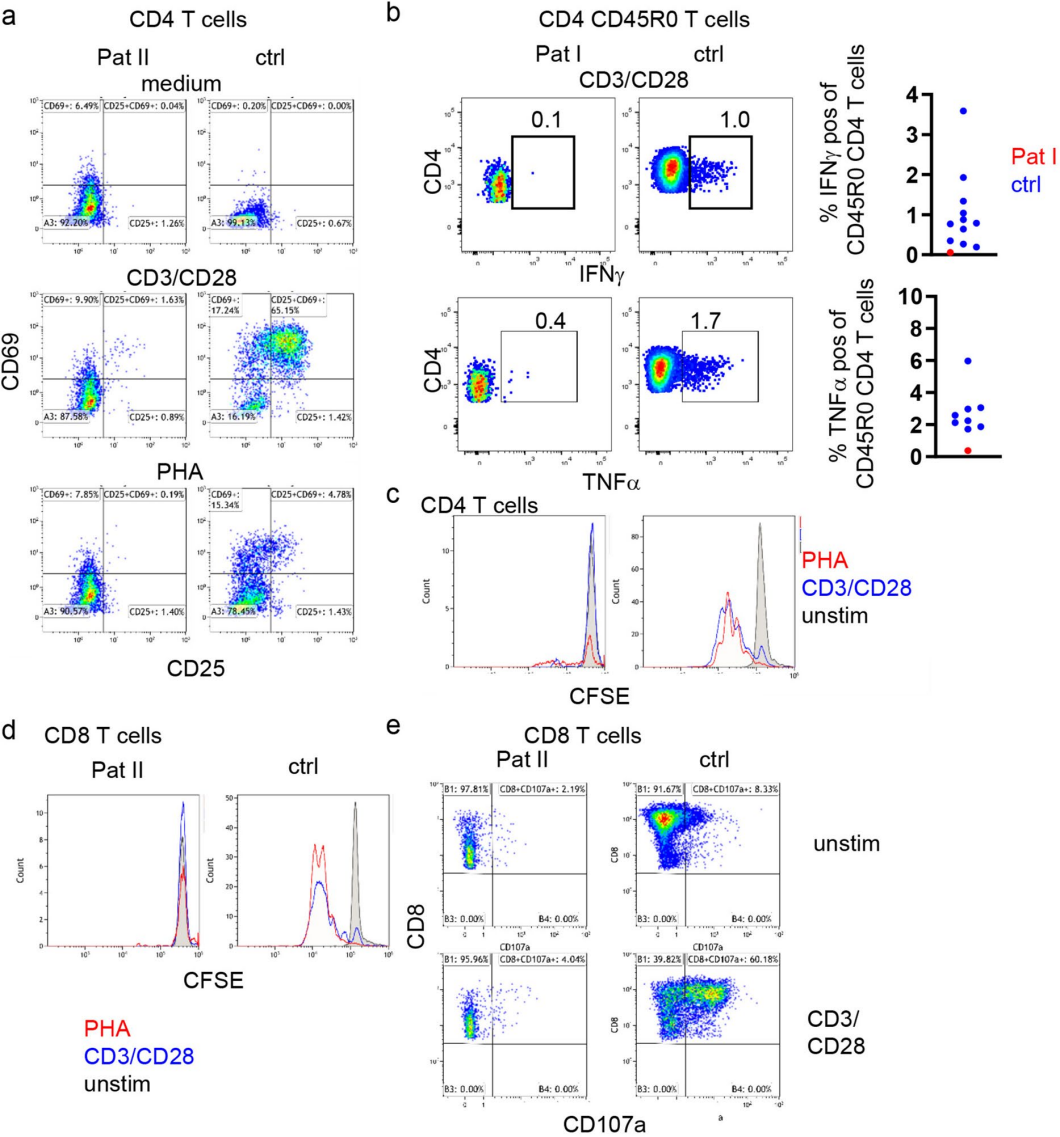
T-cell function in patients with *LCK* mutation. **a** Expression of CD69 and CD25 gated on CD4 T cells of Pat II and the respective healthy control (ctrl) unstimulated, after stimulation with anti-CD3/CD28 or PHA. **b** Representative dot plots of IFNγ and TNFα production in CD45R0/CD4 T cells after stimulation with CD3/CD28 in Pat I and one control (left) and statistical analysis of patient and 11 healthy controls. **c** Proliferation as determined by CFSE labeling in Pat II and a control unstimulated (grey), after stimulation with CD3/CD28 (blue) and PHA (red). **d** CFSE labeling gated on CD8 T cells unstimulated (black), after stimulation with CD3/CD28 (blue) and PHA (red) in patient and control. **e** CD8 cytotoxicity determined by upregulation of CD107a in Pat II and control untreated or after incubation with CD3/CD28 beads

Lymphocyte activation was evaluated in primary T cells from the patients. In accordance with the profoundly disturbed TCR signaling, the upregulation of activation markers CD69 and CD25 was impaired in patient’s CD4 T cells upon anti-CD3/CD28 stimulation (Fig. 4a). Stimulation with PHA also resulted in profoundly reduced induction of both markers, corroborating a non-redundant role for LCK in PHA-induced signaling as previously reported for T-cell lines [32]. Despite normal proportions of IFNγ- and TNFα- producing T cells after PMA/ionomycin stimulation (Fig. 2b) indicating normal differentiation of Th1 cells in vivo, the production of IFNγ and TNFα in CD45R0 CD4 T cells was severely compromised after anti-CD3/CD28 stimulation (Fig. 4b). In line, proliferation after anti-CD3/CD28 and PHA stimulation was disturbed in patients’ CD4 (Fig. 4c) and CD8 T cells (Fig. 4d). CD8 T-cell degranulation was almost abrogated in the patient’s cells (Fig. 4d). In contrast, NK-cell degranulation after stimulation with K562 cells was not affected in patient I (Supplementary Fig S4), indicating a minor role of mutated LCK in this process.

### LCKmut Function in Model T-Cell Lines

**Fig. 5:**
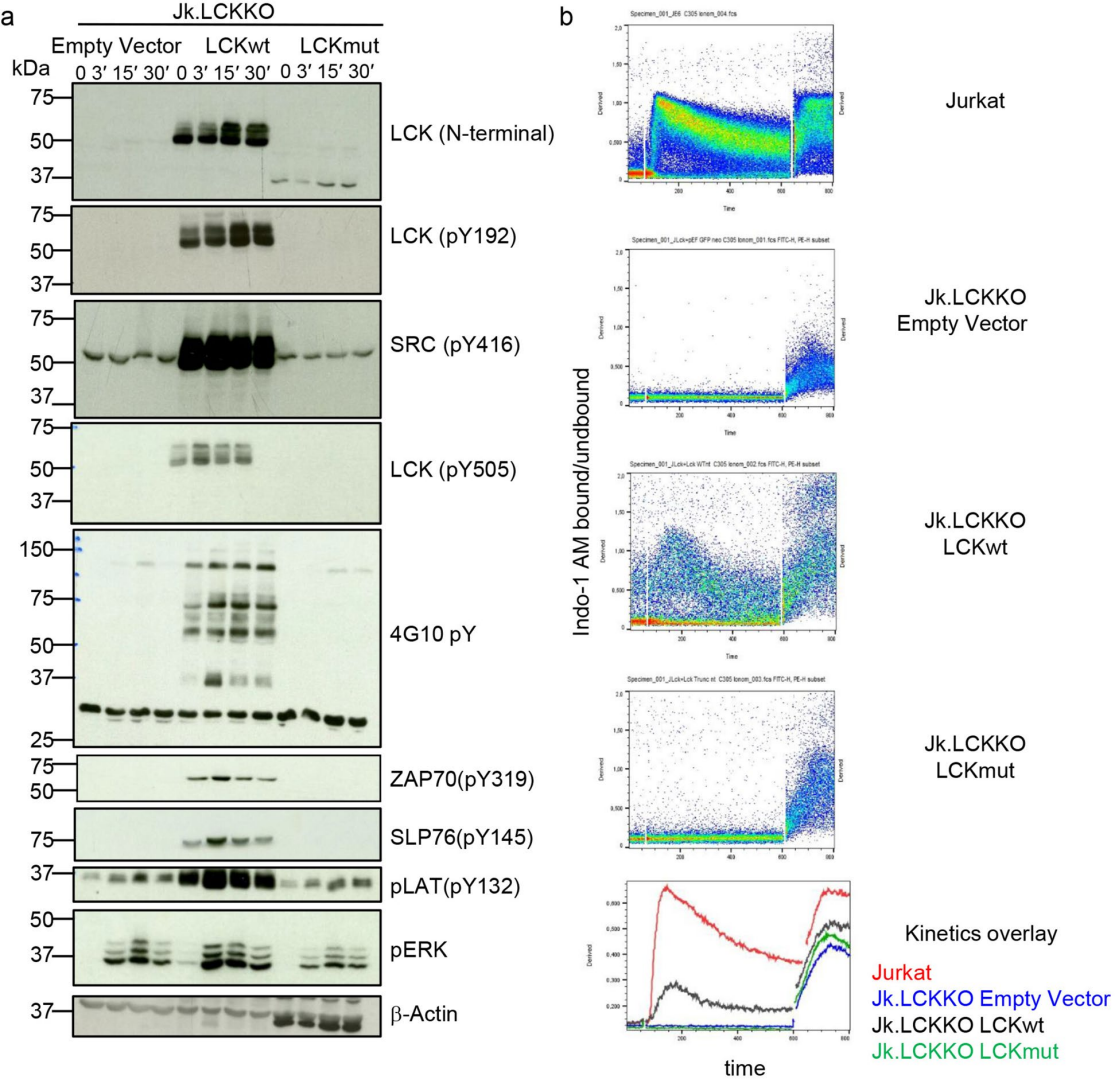
LCKmut(S377KTer14) transfected LCK-deficient T-cell lines. **a** Western blot for N-terminal LCK (as shown in Fig. 1e), pLCK (Y192), pSRC (Y416), pLCK (Y505), total tyrosine phosphorylation (4G10), pZAP, pSLP76, pLAT, pERK and β-Actin in Jk.LCKKO cells transiently transfected with an empty vector, LCKmut or LCKmut unstimulated or after stimulation with anti-CD3 for different time points as indicated. **b** Ca^2+^ mobilization in Jurkat cells, Jk.LCKKO cells transiently transfected with an empty vector, LCKwt or LCKmut. After baseline, acquisition cells were stimulated with anti-CD3. Ionomycin was added as a loading control

We next aimed to evaluate the signaling capacity of the S377KTer14 truncated LCK variant without the limitations of primary cells. To this end, LCKwt and LCKmut transiently transfected LCK-deficient Jurkat T cells (Jk.LCKKO) were compared to Jk.LCKKO cells transfected with an empty vector (Fig. 5a). As already shown in Fig. 1e but repeated for comparison, LCKmut expression was strongly reduced compared to LCKwt. Phosphorylation of Y192, Y394 (as detected by the anti-pSrcY416 antibody) and Y505 were undetectable in Jk.LCKKO cells expressing LCKmut after anti-CD3 stimulation (Fig. 5a) [33]. As expected, global tyrosine phosphorylation was strongly reduced in control Jk.LCKKO cells and cells expressing LCKmut, but reconstituted after re-expression of LCKwt as detected by developing with the 4G10 antibody (Fig. 5a), suggesting that LCKmut itself or due to its very low expression failed to propagate TCR-mediated protein phosphorylation. In line with these findings, phosphorylation of ZAP70 and SLP76 were absent after stimulation in cells expressing the LCKmut (Fig. 5a). Phosphorylation of the transmembrane adapter protein LAT was comparable to untransfected Jk.LCKKO cells but strongly reduced compared to LCKwt transfected Jk.LCKKO cells. Likewise, phosphorylation of ERK was diminished compared to LCKwt but comparable to Jk.LCKKO cells (Fig. 5a). In line with the strong reduction in TCR signaling, Ca^2+^ mobilization was abrogated in LCKmut transfected Jk.LCKKO cells after anti-CD3 stimulation, while transfection of LCKwt restored Ca^2+^ influx (Fig. 5b).

**Fig. 6:**
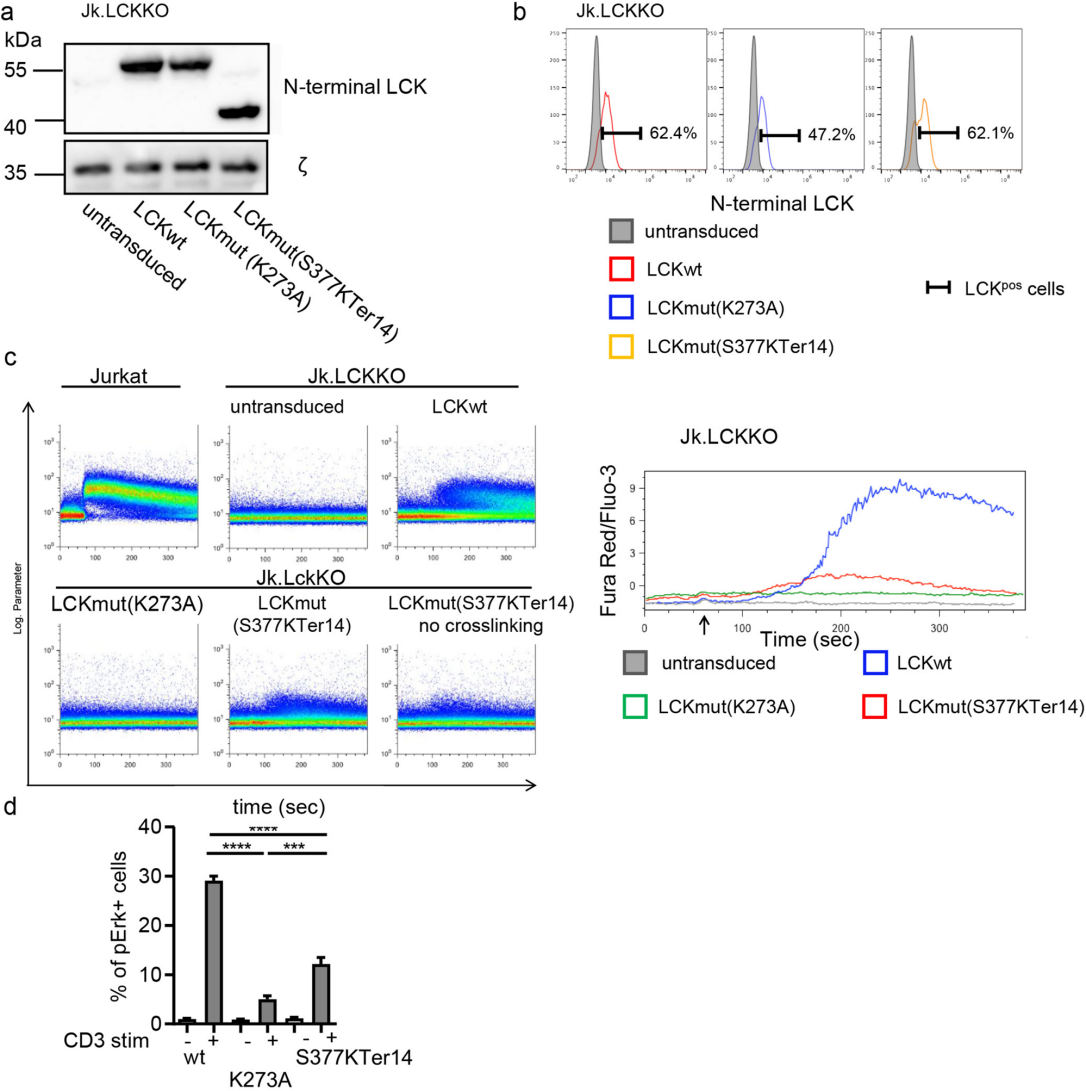
Inducible expression and analysis of LCKmut(S377KTer14) in LCK-deficient T-cell lines. **a**–**b** Jk.LCKKO cells were lentivirally transduced to express LCKwt or LCKmut under the control of a tetracycline-responsive promotor. After doxycycline-mediated induction, cells were lysed for western blot (**a**) or stained with an anti-LCK antibody recognizing the N-terminus of LCK for flow cytometry (**b**). Histograms are representative of 12 independent experiments. **c** Doxycycline-treated cells were stained with Fluo-3 and Fura-Red and stimulated with anti-CD3 alone or in combination with an anti-rabbit antibody for further crosslinking. Dot plot analysis represents Ca^2+^ influx. One representative experiment is shown out of four with similar results. **d** pERK in the indicated cells with or without anti-CD3 stimulation for 10 min as determined by flow cytometry. Statistics refer to *n* = 3 experiments. Statistical analysis was performed by one-way ANOVA analysis with Sidak’s multiple comparisons test. ***p < 0.001, ****p < 0.0001

The reduced expression of LCKmut in the patients and in the transfected cell lines (Fig. 1b and d) did not allow to investigate whether the truncated LCK protein might still support some residual function. We therefore set up a doxycycline-inducible system trying to obtain equivalent expression of LCKwt or LCKmut(S377KTer14) for the time window of the analysis in Jk.LckKO cells. As control, a kinase dead version of LCK, named LCKmut(K273A), was expressed. In this experimental setting, Doxycycline treatment for 24 h indeed resulted in similar expression levels of all three constructs (Fig. 6a and b). Under these conditions, the LCKmut(S377KTer14) was able to mount a reduced but detectable Ca^2+^ response after anti-CD3 stimulation, which was undetectable in kinase dead LCKmut(K273A) cells (Fig. 6c). Similarly, anti-CD3 stimulation resulted in a reduced phosphorylation of ERK in LCKmut(S377KTer14) cells when compared to wild type, yet increased when compared to LCKmut(K273A) (Fig. 6d). Thus, LCKmut(S377KTer14) itself appears to be capable of supporting some residual signaling events downstream of the TCR when expressed at sufficient levels in Jurkat cells.

## Discussion

Here, we report two patients harboring a novel homozygous nonsense mutation in *LCK* leading to the reduced expression of a truncated form of LCK. Beside the first patient with CID caused by a homozygous missense mutation [18], two patients with altered expression of LCK due to an alternatively spliced exon 7 have been described [34, 35]. Without reporting a specific mutation, both patients — one diagnosed with SCID and the other with common variable immunodeficiency and CD4 lymphopenia — seemed to be heterozygous for the mutations and showed largely normal TCR signaling.

The mutant LCKmut(S377KTer14) protein lacks two critical tyrosine residues regulating LCK activity: Y394 is crucial for boosting LCK kinase activity; Y505 mediates the autoregulation of LCK conformation [36]. The previously reported missense mutation L341P, which is also located in the kinase domain, similarly affected LCK protein stability, leading to reduced protein expression and lack of kinase activity [18]. However, in contrast to the L341P mutation, the LCKmut(S377KTer14) described here lacks the residue Y505, theoretically allowing the truncated protein to adopt a constitutively “open” conformation that might still function as an adaptor protein employing the SH3 and/or SH2 domains. These domains can mediate interactions with other signaling proteins like CD3ε via the recently described RK-motif in LCK’s SH3 domain [12], and with tyrosine phosphorylated proteins such as the TCR itself or ZAP70 via LCK’s unoccupied SH2 domain. This potential interaction with ZAP70 was however not associated with a detectable anti-CD3-induced phosphorylation at Y192 [26] in LCKmut(S377KTer14) in our transfection system. Phosphorylation of Y192 is relevant for the co-localization of LCK with CD45 and the TCR [26, 33]; thus, the lack of phosphorylation may contribute to the observed aberrant subcellular localization of the truncated protein despite an intact N-terminus domain anchoring LCK to the plasma membrane in the truncated LCKmut [37].

All three patients with homozygous *LCK* mutations and knock out mice showed decreased CD4 and CD8 expression [18, 29]. As the N-terminal motif in the UR of LCK for CD4 and CD8 interaction [38, 39] is preserved in these patients, diminished LCK expression most likely account for reduced expression of both co-receptors [40]. Especially, the reduced expression of CD4 but not CD8 seems to be unique to LCK deficiency and may therefore serve as a valuable diagnostic hint in patients with CID, whereas altered expression of CD8 can also be a regulatory mechanism during chronic or acute activation [41].

In primary T cells from our patients and in the transiently LCKmut(S377KTer14) transfected Jk.LCKKO cells, the levels of truncated LCK were strongly reduced, and TCR-downstream signaling was severely compromised, resulting in defective in vitro T-cell activation. These results corroborate the current comprehension of LCK biology. In particular, we demonstrated severely altered anti-CD3 induced Ca^2+^ mobilization as well as blunted phosphorylation of SLP76 and ITK in the patient’s primary T cells in line with the data from the LCK-deficient T-cell blasts shown by Hauck et al. [18]. Residual ERK phosphorylation as shown by Hauck et al. was also detectable in LCK-deficient T cell lines, indicating that some LCK kinase activity independent signaling can occur, possibly due to redundancy with other Src kinases like Fyn. Here, we have demonstrated for the first time persisting mTOR signaling in patients’ primary T cells when most TCR downstream signaling was abolished. Given the interference of specific LCK inhibitors with AKT and S6 phosphorylation, the observed mTOR phosphorylation might suggest alternative LCK- and AKT-independent mTOR activation [42, 43]. Alternatively, the residual expression of the truncated, most likely kinase-inactive LCK protein may suffice to allow for mTOR activation but not of other signaling pathways. Due to constitutively active PI3K in Jurkat T-cell lines [44], we were not able to address this question in our transfection or transduction systems.

IEI affecting different components of the TCR signalosome encompass severe, partly overlapping immunological abnormalities [3]. All three homozygous LCK-mutated patients presented with prominent CD4 and progressive CD8 lymphopenia as well as increased proportions of γδT cells. CD8 memory T cells may be less dependent on intact LCK signaling [45]. Additionally, viral infections, especially by CMV, may contribute to an even more severe lymphopenia and altered composition of the subpopulations with an enrichment of CD8^pos^ γδT cells [46], and terminally differentiated CD8 effector T cells [47, 48]. Both of our patients had severely reduced naïve T-cell populations, but nearly normal proportions of circulating Tregs, TFH, and TH1, TH2, and TH17 cells. This demonstrates that the in vivo differentiation of these memory populations is possible despite impaired TCR signaling. In mice, it has been demonstrated that FYN can partly replace LCK in thymic development [49], allowing for a low number of T cells to develop. This may also be true in humans, but it remains to be seen which molecules are replacing LCK during the peripheral differentiation of the various memory, effector, or regulatory subsets. However, these mechanisms seem insufficient to compensate for reduced LCK activity during acute TCR activation, as this process is severely compromised in patients’ T cells. Alternatively, or additionally to the functional substitution by other Src-family kinases, a preserved residual biological activity of the truncated LCKmut, possibly as a docking partner, cannot be completely ruled out, given the results obtained when the truncated LCK was analyzed in the time window of (unnaturally) high expression LCK in our inducible model.

The severely impaired function, including the lack of T-cell cytotoxicity, predisposes LCK patients to an increased susceptibility for a wide variety of pathogens, even though NK-cell cytotoxicity was preserved [50, 51]. Our patients suffered from early onset recurrent infections, suspected *Pneumocystis jirovecii* pneumonia and viremia, indicating the profound CID in both children, but unlike the first-described patient [18] not with prominent signs of autoimmunity.

When comparing the clinical phenotype with other CID due to TCR signaling defects, LAT and ZAP70 deficiency present with the strongest tendency towards autoimmunity [5, 8, 52]. Patients with deficiencies in ITK and SLP76 presented with recurrent and opportunistic infections, but autoimmune manifestations have been reported in single patients [6, 7, 20, 53, 54]. There is no single clinical manifestation which can distinguish the different forms of CID. Immunologically, all but ZAP70 present with early onset progressive CD4 lymphopenia. In all these patient, CD8 counts are variable but typically low [6–8, 20, 52, 53, 55]. In patients with LAT deficiency, CD4 T cells were biased towards a TH2 phenotype as in our LCK-deficient patients [8], whereas patients with ITK deficiency showed reduced TH17 but increased TH1 responses [56]. The relative expansion of γδT cells in LCK-deficient patients has also been observed in LAT and SLP76 deficiency but has not been addressed in ITK or ZAP70 deficiency [7, 8]. Similar to a SLP76-deficient patient [54], the TRG repertoire showed reduced diversity, higher clonality, and preferential gene utilization in our patients. Although preferential *TRGV8* gene utilization was observed in LCK and SLP76-deficient patients, respectively, all showed lower levels of *TRGV10* in the peripheral blood. The importance of the specific TRG-γδT cells are not yet known in humans; however, in mice, it is known that certain γδT cells acquire certain effector functions according to expression of respective *TRGV* genes [57] and viral infection can further skew TRGV usage as previously shown for CMV [58].

Given the low numbers of patients with the different mutations causing CID, conclusions need to be still taken with care, but future evaluation of these patients may reveal new insights into the role of specific molecules in fundamental processes as positive and negative selection, differentiation of regulatory, memory, and effector cells. Our findings increased our comprehension of the impact of LCK deficiency in humans, which was previously limited to a single patient. The consistent alterations in LCK-deficient T cells corroborates common clinical, immunological, and molecular manifestations of this rare immunodeficiency including the potential diagnostic hallmark of reduced CD4 expression but absence of autoimmunity until HSCT in one of our patient. Here, we demonstrated for the first time normal mTOR activation and preserved differentiation of all memory T-cell populations despite severely disturbed cellular activation and function.

## Conclusion

In LCK-deficient patients with combined immunodeficiency, low CD4 expression serves as a specific hallmark. The defect strongly impairs T-cell function despite residual TCR signaling and T-cell differentiation. Our findings expand the previous report on one single patient on the central role of LCK in human T-cell development and function.

### Supplementary Information

Below is the link to the electronic supplementary material.

Supplementary file1 (DOCX 1353 KB)

Supplementary file2 (DOCX 14 KB)

## Data Availability

All data generated or analyzed during this study are included in this published article and its supplementary information files.
