# Supplementary material for: Combined Immunodeficiency Caused by a Novel Nonsense Mutation in LCK
Source: J Clin Immunol. 2023 Dec 19;44(1):4. doi: 10.1007/s10875-023-01614-4 (PMC10730691; doi:10.1007/s10875-023-01614-4)
Supplement: Supplementary file 1 — Supplementary file1 (DOCX 1353 KB) [file 10875_2023_1614_MOESM1_ESM.docx]

**Appendix**

**Supplementary Figures and Figure Legends**

**
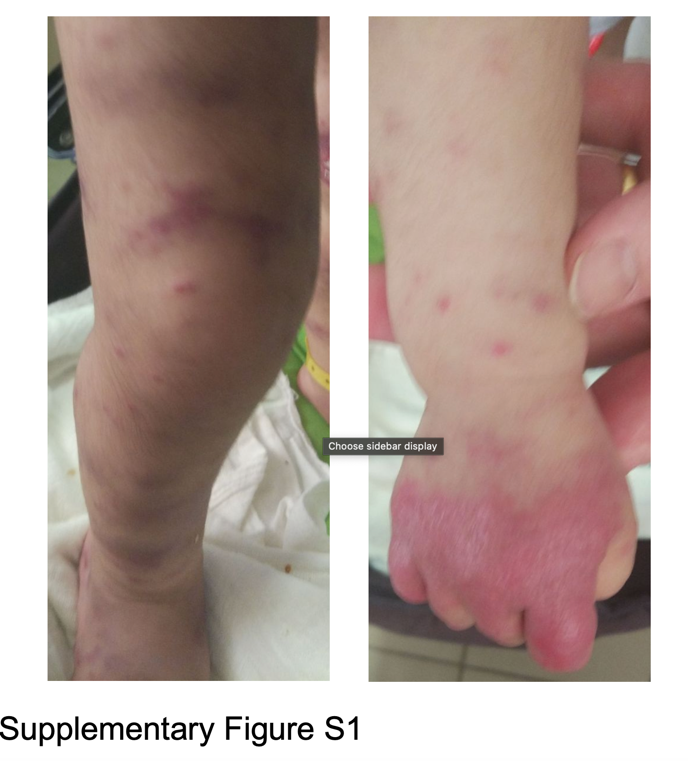
**

**Supplementary Fig. S1 Clinical presentation of the skin lesions**

Pat II presented with recurrent erythematous ulcerative skin plaques and nodules on the palm and leg.

**
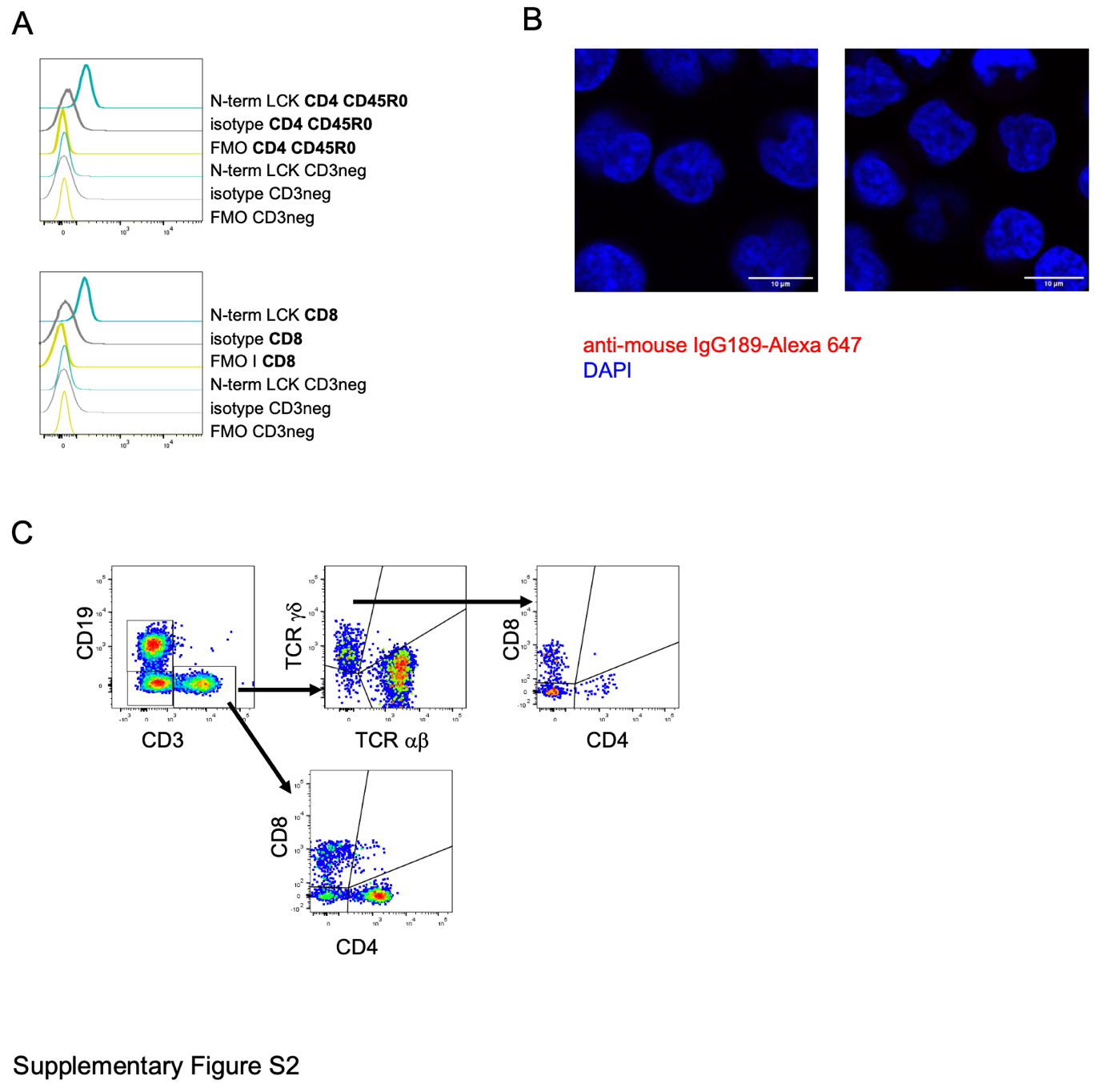
**

**Supplementary Fig. S2 Supplementary flow cytometric and microscopy data**

A) Histogram of N-terminal LCK staining, isotype control and FMO (fluorescence minus one) in CD45R0 CD4 T cells or CD8 T cells compared to CD3neg cells from the same healthy donor as shown in Figure 1c. B) Secondary antibody staining for LCK transduced Jk.LCKKO cells and DAPI as shown in Figure 1f. C) Gating strategy and example for γδ T cell staining in Pat I and definition of CD8pos and DN T cells as shown in Figure 2a.

**
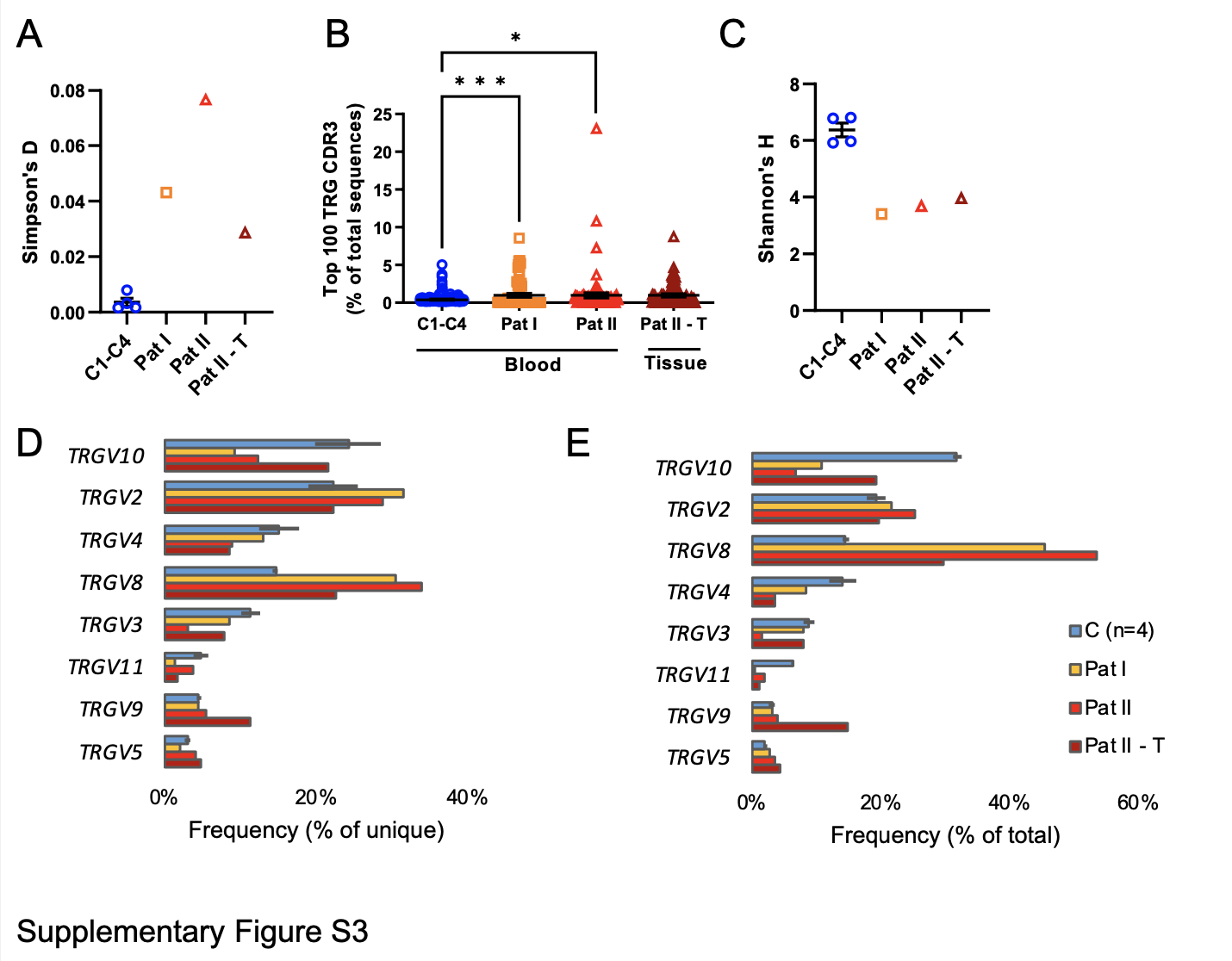
Supplementary Fig. S3 TRG repertoire in LCK patients**

The unique number of sequences corresponds to the number of unique clonotypes and the total number of sequences reflects the total number of circulating cells. The unique number of sequences in Pat I and Pat II were 257 and 520 sequences, respectively, compared to 2,066-4,260 sequences in healthy pediatric controls. The total number of sequences were 7,630 and 18,562 sequences, respectively, compared to 78,178-159,824 sequences in controls. Comparable to the peripheral blood, the unique and total sequences from Pat II skin biopsy were 428 and 14,165, respectively. Scatter dot plot presenting the (a) Simpson’s D diversity index in patient samples and controls (n=4). (b) Scatter dot plot presenting the frequency of the top 100 most abundant clones in patient samples and pediatric controls. (c) Shannon’s H diversity index in patient samples and controls (n=4). The p-values are for one-tailed t-test with Welch’s correction and whiskers present standard error (±SE). (d) and (e) Bar graph representing the average of four pediatric controls (±SE) and patients’ samples from peripheral blood and skin biopsy for the unique (d) and total (e) sequences. *TRGV8* gene was profoundly utilized in both patients’ γδ T cells in peripheral blood or tissue, both in unique and total sequences. The higher frequencies of *TRGV8*^+^ sequences in total sequences is indicative of its involvement in clonal expansions. Furthermore, *TRGV10*^+^ sequences of peripheral blood were substantially lower in both patients in unique and total sequences and *TRGV10*^+^ sequences of skin biopsy were markedly less utilized only in total sequences.

**
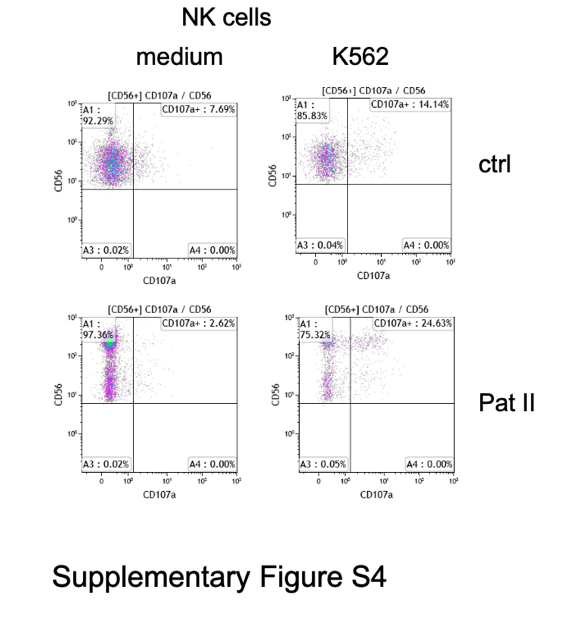
**

**Supplementary Fig. S4 NK cell degranulation is normal in LCK patients**

CD107a expression in one ctrl and Pat II after incubation with K562 target cells or unstimulated which was unremarkable in patient’s CD56pos NK cells.
