# Supplementary material for: Combined Immunodeficiency Caused by a Novel Nonsense Mutation in LCK
Source: J Clin Immunol. 2023 Dec 19;44(1):4. doi: 10.1007/s10875-023-01614-4 (PMC10730691; doi:10.1007/s10875-023-01614-4)
Supplement: Supplementary file 2 — Supplementary file2 (DOCX 14 KB) [file 10875_2023_1614_MOESM2_ESM.docx]

**Appendix**

**Supplementary Information – Material and Methods**

*Cell isolation and cultivation of primary cells*

Peripheral blood mononuclear cells (PBMCs) were isolated from EDTA blood by Ficoll density gradient centrifugation following standard protocols. PBMCs were cultivated in IMDM (Invitrogen) containing 10% FCS.

*Whole exome sequencing*

WES was performed with a SureSelect Human All Exon (Agilent, Santa Clara, CA, USA), and sequenced on a HiSeq2500 platform (Illumina, San Diego, CA, USA). Read alignment and variant calling were performed with DNAnexus (Palo Alto, California, USA). The LCK mutation was validated by using dideoxy Sanger sequencing in the patients, parents and two siblings of P2 and healthy control (HC). Data were evaluated using Sequencer software (version 5.0; Gene Codes Corporation).

*Microscopic analysis of transfected Jk.LCKKO cells*

Following transfection and overnight incubation, the transfectants were washed twice in PBS, 3x10^4^ cells were transferred onto adhesion glass slides and fixed for 15 min at RT in 1% PFA/0,025% Glutaraldehyd. Cells were permeabilized for 10 min using PBS 0.2% Triton X100. Slides were washed 3 times with PBS, blocked for 30 min using PBS 1% BSA and stained with anti-LCK antibody (3A5, Santa Cruz) for 45 min at RT. Subsequently slides were washed and stained with anti-mouse IgG189-Alexa 647 (Thermo Fischer Scientific) and DAPI. Slides were washed and embedded into Vectashield H-1000 (Vector Laboratories Inc, Burlingame, Ca). Slides were visualized using an inverted Confocal Microscope System Leica SP8 (Leica Mannheim, Germany) equipped with a Plan Apo 63x/1.4 oil objective and controlled by the LASX software (Leica). To avoid bleed-through between the different spectral channels, sequential unidirectional scanning was performed at 600 Hz using the following settings: sequence 1: excitation 488 nm, emission 495–545 nm and excitation 633nm, emission 652-705 nm combined with transmitted light detection; sequence 2: excitation 405 nm, emission 431-475 nm. Sequences were altered between lines. Voxel size was adjusted to 92 nm × 92 nm × 230 nm (dx, dy, dz). Images of the individual channels were pseudo colored: GFP (excitation 488 nm) in green (channel not shown), Alexa 647 (excitation 633nm) in red, DAPI (excitation 405 nm) in blue. Single planes out of the data stacks were analyzed using ImageJ software (<https://imagej.nih.gov/ij/index.html>).
